# Do home adaptation interventions help to reduce emergency fall admissions? A national longitudinal data-linkage study of 657,536 older adults living in Wales (UK) between 2010 and 2017

**DOI:** 10.1093/ageing/afab201

**Published:** 2021-10-18

**Authors:** Joe Hollinghurst, Helen Daniels, Richard Fry, Ashley Akbari, Sarah Rodgers, Alan Watkins, Sarah Hillcoat-Nallétamby, Neil Williams, Silviya Nikolova, David Meads, Andy Clegg

**Affiliations:** Swansea University Medical School, Swansea University, Swansea, UK; Swansea University Medical School, Swansea University, Swansea, UK; Swansea University Medical School, Swansea University, Swansea, UK; Swansea University Medical School, Swansea University, Swansea, UK; University of Liverpool, Department of Public Health, Policy and Systems, Liverpool, UK; Swansea University Medical School, Swansea University, Swansea, UK; Swansea University Medical School, Swansea University, Swansea, UK; Head Office, Care & Repair Cymru, Cardiff UK; Academic Unit of Ageing and Stroke Research, University of Leeds, Leeds, UK; Academic Unit of Ageing and Stroke Research, University of Leeds, Leeds, UK; Academic Unit of Ageing and Stroke Research, University of Leeds, Leeds, UK

**Keywords:** older people, falls prevention, frailty, falls

## Abstract

**Background:**

falls are common in older people, but evidence for the effectiveness of preventative home adaptations is limited.

**Aim:**

determine whether a national home adaptation service, Care&Repair Cymru (C&RC), identified individuals at risk of falls occurring at home and reduced the likelihood of falls.

**Study Design:**

retrospective longitudinal controlled non-randomised intervention cohort study.

**Setting:**

our cohort consisted of 657,536 individuals aged 60+ living in Wales (UK) between 1 January 2010 and 31 December 2017. About 123,729 individuals received a home adaptation service.

**Methods:**

we created a dataset with up to 41 quarterly observations per person. For each quarter, we observed if a fall occurred at home that resulted in either an emergency department or an emergency hospital admission. We analysed the data using multilevel logistic regression.

**Results:**

compared to the control group, C&RC clients had higher odds of falling, with an odds ratio (OR [95% confidence interval]) of 1.93 [1.87, 2.00]. Falls odds was higher for females (1.44 [1.42, 1.46]), older age (1.07 [1.07, 1.07]), increased frailty (mild 1.57 [1.55, 1.60], moderate 2.31 [2.26, 2.35], severe 3.05 [2.96, 3.13]), and deprivation (most deprived compared to least: 1.16 [1.13, 1.19]). Client fall odds decreased post-intervention; OR 0.97 [0.96, 0.97] per quarter. Regional variation existed for falls (5.8%), with most variation at the individual level (31.3%).

**Conclusions:**

C&RC identified people more likely to have an emergency fall admission occurring at home, and their service reduced the odds of falling post-intervention. Service provisioning should meet the needs of an individual and need varies by personal and regional circumstance.

## Key Points

- Home adaptations help to reduce the trajectory of future falls.
- Falls are associated with frailty severity.
- Falls prevention should be prioritised

## Introduction

Healthy ageing, prevention and long-term-care needs are key challenges for policy makers, planners, commissioners and providers seeking to ensure sustainability of health and social care services internationally [1–3]. Projections of future care needs for older people in England and Wales indicate considerable challenges in this area, with an anticipated increase of 25% by 2025 [4].

Falls are common among older people and cause increased morbidity, mortality and use of health care services [5]. Highlight notices from the World Health Organisation and the NHS identify the need for the prevention of falls [6, 7]. It is estimated that falls cost the NHS more than £2.3 billion per year, with 30% of people aged 65+ and 50% of people aged 80+ falling at least once per year [8].

Older people typically prefer to remain living independently in their own home for as long as possible rather than transition to a long-term care environment, and independent home-living supports overall health and wellbeing [9]. Home adaptation services are an example of one approach that aims to support independent home-living and reduce falls at home, but there is insufficient high quality evidence to support widespread commissioning [10]. Care & Repair Cymru (C&RC) are a national charity in Wales (UK) who provide home adaptation interventions and advice to support people to live safely in their own homes. Individuals can be referred to C&RC via a healthcare professional or by self-referral (by the individual, or by their family/friends). C&RC provide advice free of charge, this can include helping with grant applications to fund home adaptations and the subsequent management of contractors. For more details on the services C&RC provide, please see [11]. The charity operates via 13 regional agencies that cover all 22 local authority areas in Wales and are partly funded by the Welsh Government. Falls prevention is a national priority in Wales [12], and C&RC are a partner, and provide the ‘chair’ of the Improvement Cymru Multiagency Falls Collaborative (National Prudent Healthcare Falls Prevention Taskforce) set up by Public Health Wales with endorsement from Welsh Government, seeking to reduce falls in the community [13].

Delivering services to those at highest risk is key to reducing falls [14]. Factors found to put people at risk of falls include increasing age [15], previous falls [16], cognitive impairment [17] and frailty [18] amongst others, as shown in [19]. Given that falls prevention strategies are often dependent on local and regional commissioning priorities [20], identifying whether spatial variation in fall risk exists will allow for more efficient planning, service prioritisation and potentially reduce health inequalities.

The use of existing anonymised routinely collected longitudinal data can help to provide access to large-scale data for studies [21] and provide robust evidence for commissioning decisions and policy [22]. In this study, we linked administrative and electronic health records to investigate fall outcomes following home adaptation interventions. Our key objectives were to (1) determine if home adaptation interventions led to a reduction in falls resulting in emergency admissions to hospital or an accident and emergency department, and (2) investigate if there were differences in fall risk based on area.

## Methods

### Data sources

Our cohort was created using data held within the Secure Anonymised Information Linkage (SAIL) Databank [23–25]. The SAIL Databank is a privacy protecting Trusted Research Environment (TRE) and contains anonymised records, with the anonymisation performed by a trusted third party (TTP)—the NHS Wales Informatics Service (NWIS). The SAIL Databank has a unique individual anonymised person identifier known as an Anonymous Linking Field (ALF) and unique address anonymised identifier known as a Residential Anonymous Linking Field (RALF) [26] that are used to link between data sources at individual and residential levels, respectively. Individual linking fields, nested within residences, are both contained in the anonymised version of the Welsh Demographic Service dataset (WDSD) replacing the identifiable names and addresses of people who are registered with a free-to-use General Practitioner (GP) service. We used the Patient Episode Database for Wales (PEDW) and the Emergency Department Data Set (EDDS) for details on emergency hospital admissions and accident & emergency department attendances respectively. Intervention data covering April 2009—December 2017 were provided by C&R, with anonymisation and linkage to the SAIL Databank via the TTP.

### Study design

We used longitudinal anonymised electronic health records (EHRs) and administrative data from the SAIL Databank to create a controlled non-randomised intervention cohort using data linkage.

### Setting

Individuals in Wales aged 60+ years who were registered with a general practice submitting data to the SAIL Databank, stored in the Welsh Longitudinal General Practice (WLGP) primary care data.

### Participants

We used demographic and primary care data collected from 1 January 2010 to 31 December 2017 to define our cohort (*N* = 657,536). SAIL currently receives data from 80% of general practices in Wales, which contains ~2.4 billion primary care events [27]. General practice data from 1 January 2000 to 31 January 2017 were used to define the level of frailty of individuals, by calculating the electronic Frailty Index (eFI) for a 10 year period prior to the start of each quarterly interval [28, 29]. We used intervention data from C&RC, mortality data from the Office for National Statistics (ONS), and demographic data from WDSD from 1 January 2010 to 31 December 2017. We included the 2014 Welsh Index of Multiple Deprivation (WIMD) quintile at the start of each quarterly interval as a measure of socioeconomic status [30] using the 2011 version of the Lower-layer Super Output Area (LSOA).

### Care&Repair Cymru clients—Intervention group

We anonymously linked the C&RC register to the SAIL Databank using a split file process. This included the dates of C&RC home adaptation intervention and the type of intervention. Our intervention cohort consisted of older people who received a C&RC intervention between 1 January 2010 and 31 December 2017 (*N* = 123,729). We collaborated with C&RC to identify categories of interventions with the primary purpose of fall prevention; detailed information is contained in Supplementary Table S1. The falls prevention sub-types and examples of the interventions include:

- Care&Repair Cymru client (yes/no); *N* = 123,729 (100%).
- Falls on a level; *N* = 72,151 (58.3%): e.g. grab rails, floor coverings, handrails.
- Falls on stairs; *N* = 27,595 (22.3%): e.g. bannister, stair rail, stairlift.
- Falls between levels; *N* = 27,349 (22.1%): e.g. step lift, external rails, ramp.
- Falls in the bathroom or bedroom; *N* = 11,716 (9.5%): e.g. level access shower, bathroom redesign, hoist.
- Cold homes; *N* = 3,201 (2.6%): e.g. boiler repairs, central heating, heating repairs.

### Non-clients—Control group

Our control cohort was created by randomly assigning an intervention date from people receiving a C&RC service to those who did not. To distinguish between groups, we included an indicator for if and when someone received an intervention.

### Electronic frailty index

The eFI is based on the internationally established cumulative deficit model of frailty, and assigns a frailty index score to an individual using 36 variables from primary care GP data including falls, symptoms, signs, diseases, disabilities and abnormal laboratory values, referred to as deficits [31]. The eFI score is the number of deficits present, expressed as an equally weighted proportion of the total. An individual with a single deficit would be assigned an eFI of 1/36 (0.03); another with nine deficits would be assigned an eFI of 9/36 (0.25). The eFI score is used to categorise individuals as: fit (eFI value of 0–0.12), mild (>0.12–0.24), moderate (>0.24–0.36) or severe frailty (>0.36) [28, 29]. We calculated the eFI on the start date of each quarter using a maximum window of 10 years of previous primary care data.

### Main outcome

The main outcome of interest was a fall-related emergency attendance at an Emergency Department (ED), or emergency admission to hospital. In both cases, we restricted to falls occurring at home. We combined both to create a binary indicator of falls for use in our analysis. If an individual attended the ED and was then admitted to hospital in the same quarter, this was counted as a single event. We have specified the codes used to identify falls in the Supplementary material, section falls coding.

### Dataset design

Quarterly observation periods were centred around the intervention (index) date (the randomly assigned comparator for the control, non-C&RC clients) for each individual in the study. The time quarters were labelled from −20 to +20, where the start date for quarter 0 was the date an intervention was received. We observed individuals for up to 5-years pre- and post-intervention date. Individuals were censored in the dataset for quarters after death and address moves. We included a binary indicator for a fall-related emergency admission at home to either an ED or a hospital for each time quarter. Another binary variable indicated C&RC client status and intervention categories. Client status and an intervention category indicator were changed to ‘yes’ in the dataset when an individual had received a C&R intervention or the specified intervention category. For example, a C&R client would have ‘yes’ recorded in the C&R intervention variable for time quarters 0–20 (or up to the censor point), and ‘yes’ recorded for the ‘Falls on a level’ when an intervention included in the ‘Falls on a level’ category was received.

Age and sex were fixed at the intervention date as baseline risk variables. The eFI and WIMD varied with time and were calculated at the start of each quarter. We also included the 13 C&RC regions in the dataset. The composition of the C&RC regions in relation to the Welsh local authorities is detailed in the Supplementary material, section C&RC regions (Supplementary Table S2).

### Statistical methods

Multilevel logistic regression was used to analyse the repeated-measures data. We used a stepwise approach to assess the influence of including additional variables in the models. The dependent variable was the binary indicator for an ED attendance or hospitalisation following a fall at home. The time quarter (−20 to 20) and age (60+) were included as continuous variables. The C&R client status (yes/no), intervention sub-groups (yes/no), sex (male/female), eFI (fit, mild, moderate, severe) and WIMD (1. Least deprived to 5. Most deprived) were included as categorical variables. Interaction terms were calculated between the time period and both the C&RC client status and intervention sub-types. Age and sex were fixed using their values at time quarter 0. The C&R client status, intervention sub-types, WIMD and eFI were time varying and were updated at each quarter. The following hierarchy was used in the modelling: 1. observation, 2. individual, 3. C&RC region. Quarters with missing data were removed from the dataset, this occurred when residential information was not available. To quantify the variance at individual and regional levels, random effects were added. We estimated the variance partition coefficients at the individual and C&RC regional levels using the latent variable method [32]. R version 4.0.0 and R2MLwiN [33] were used for all analyses.

## Results

### Descriptive data and regional variation

The dataset had a total of 22,016,986 quarterly observations, with a maximum of 41 observation periods per individual (5-years pre and post intervention, plus the intervention quarter), and an average of 33 complete quarters per person as individuals were censored after their death date. Descriptive data for the cohort as a whole, along with the stratification for C&RC clients and control groups are presented in Table 1. The table includes values for one quarter (3-months) prior to the inception date along with data for the inception date and 5-years post inception for all groups. Extended quarterly data for the study period are available in the Supplementary material, Supplementary Tables S3–S5.

**Table 1:** Descriptive data for the total cohort, Care & Repair Clients and control group (non-clients)

	Combined			Control group (non-clients)			Care&Repair clients		
Quarterly period	−1 (3 months prior)	0	20 (5 years post)	−1 (3 months prior)	0	20 (5 years post)	−1 (3 months prior)	0	20 (5 years post)
Individuals (*N*)	655,671	657,536	243,294	532,492	533,807	207,647	123,179	123,729	35,647
Falls (*N*)	9,858	4,758	1,822	2,025	2,202	1,119	7,833	2,556	703
Falls rate (%)	1.50%	0.72%	0.75%	0.38%	0.41%	0.54%	6.36%	2.07%	1.97%
Mean age	72	72	69.81	70.6	70.6	68.82	78.03	78.04	75.61
S.D. Age	8.79	8.8	7.6	8.25	8.25	7.07	8.52	8.52	8.01
Sex—Male	46.25%	46.25%	45.39%	48.21%	48.22%	47.93%	37.76%	37.77%	30.60%
Sex—Female	53.75%	53.75%	54.61%	51.79%	51.78%	52.07%	62.24%	62.23%	69.40%
eFI—Fit	53.68%	52.70%	48.07%	59.34%	58.71%	52.69%	29.25%	26.77%	21.19%
eFI—Mild	32.48%	32.66%	35.04%	30.38%	30.65%	34.58%	41.57%	41.32%	37.67%
eFI—Moderate	11.18%	11.73%	13.24%	8.49%	8.74%	10.51%	22.84%	24.65%	29.13%
eFI—Severe	2.65%	2.91%	3.65%	1.80%	1.91%	2.22%	6.35%	7.26%	12.00%
WIMD—1.Least deprived	22.49%	22.49%	23.11%	23.18%	23.17%	23.96%	19.51%	19.53%	18.13%
2	19.78%	19.80%	19.87%	19.86%	19.87%	19.94%	19.45%	19.51%	19.41%
3	21.13%	21.14%	21.05%	20.95%	20.96%	20.81%	21.92%	21.94%	22.41%
4	19.72%	19.71%	19.52%	19.24%	19.23%	19.01%	21.78%	21.75%	22.47%
5.Most deprived	16.88%	16.86%	16.46%	16.77%	16.77%	16.27%	17.34%	17.27%	17.58%

**Table 2:** Multilevel logistic regression ORs for emergency related falls admissions occurring at home

	Regression coefficients	Odds ratios (95% Confidence intervals)
Intercept	−11.291 (−11.597, –10.984)	0
Time period (Quarter)	0.039 (0.038, 0.040)	1.040 (1.039, 1.040)
Age	0.066 (0.065, 0.066)	1.068 (1.067,1.069)
eFI—Mild	0.453 (0.436, 0.469)	1.572 (1.546,1.599)
eFI—Moderate	0.836 (0.816,0.856)	2.306 (2.261, 2.353)
eFI—Severe	1.114 (1.086, 1.142)	3.047 (2.962, 3.133)
Sex (Female)	0.366 (0.351, 0.381)	1.441 (1.42, 1.463)
Deprivation (WIMD, reference 1. Least Deprived)		
2.	0.023 (−0.001, 0.046)	1.023 (0.999, 1.047)
3.	0.063 (0.040, 0.086)	1.065 (1.041, 1.089)
4.	0.123 (0.100, 0.145)	1.130 (1.105, 1.156)
5. Most deprived	0.149 (0.126, 0.172)	1.161 (1.134, 1.188)
*Care&Repair interventions*		
Care&Repair client	0.659 (0.625, 0.692)	1.933 (1.869, 1.999)
Bathroom & bedroom	0.039 (−0.051, 0.128)	1.040 (0.951, 1.137)
Between levels	−0.096 (−0.156, –0.036)	0.908 (0.856, 0.964)
Cold prevention	−0.325 (−0.529, –0.121)	0.723 (0.589, 0.886)
Falls on a level	0.223 (0.180,0.266)	1.250 (1.197, 1.305)
On the stairs	0.029 (−0.029, 0.087)	1.029 (0.971, 1.091)
*Interactions*		
Time period (Quarter): Care&Repair Client	−0.034 (−0.037, –0.030)	0.967 (0.964, 0.97)
Time period (Quarter): Bathroom & bedroom	0.008 (0.001, 0.016)	1.008 (1.001, 1.016)
Time period (Quarter): Between levels	0.009 (0.003, 0.014)	1.009 (1.003, 1.014)
Time period (Quarter): Cold prevention	0.026 (0.009, 0.042)	1.026 (1.01, 1.043)
Time period (Quarter): Falls on a level	0.000 (−0.005, 0.004)	1.000 (0.995, 1.004)
Time period (Quarter): On the stairs	0.006 (0, 0.011)	1.006 (1, 1.011)
*Random effects*		
Individual level variance	1.635 (1.61, 1.659)	
Care&Repair Region level variance	0.302 (0.07, 0.535)	
Observations	22,016,986	22,016,986
Individuals	657,536	657,536
Care&Repair regions	13	13

**Figure 1:**
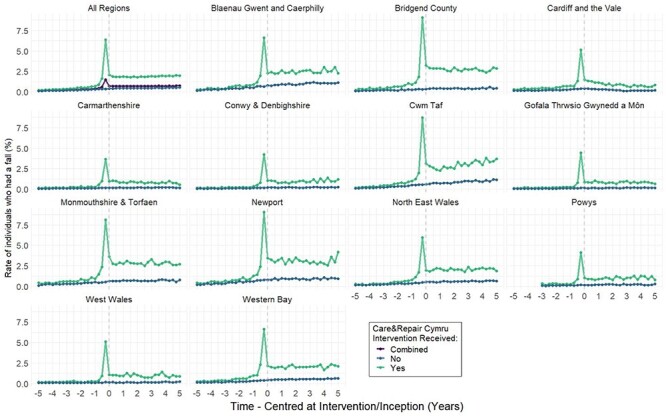
Unadjusted rate (%) of individuals who had a fall per quarter. Each sub-figure represents a C&RC region, stratified by individuals receiving a C&RC service (green) or not (blue). The combined rate is shown in the sub-figure for all regions (purple).

Figure 1 shows the falls in each quarter as a proportion of the total number of people in each regional subgroup stratified by C&R-clients and the control groups. In the plot for all regions, the combined cohort shows a steady increase in the rate of people who had a fall, with a distinct peak at the index date (quarter 0). A subset of data is presented for Powys due to the information governance concerns of small counts in this region with sparse population, but data for all time periods were included in the regression models.

### Regression results

**Figure 2:**
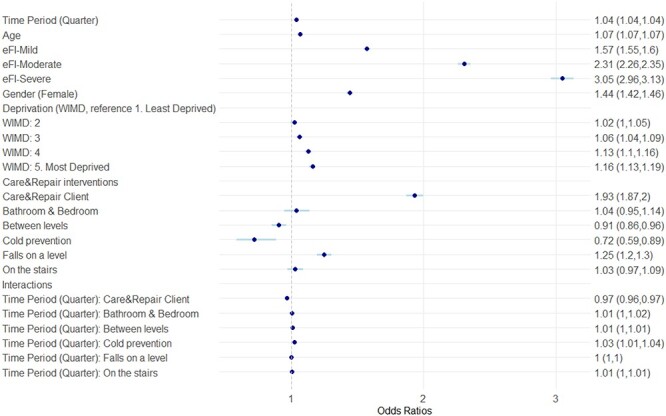
ORs and 95% confidence intervals for the fixed effects from the multilevel logistic regression model. ORs < 1 indicated reduced odds of an emergency admission for a fall, ORs > 1 indicate increased odds of an emergency admission for a fall.

**Figure 3:**
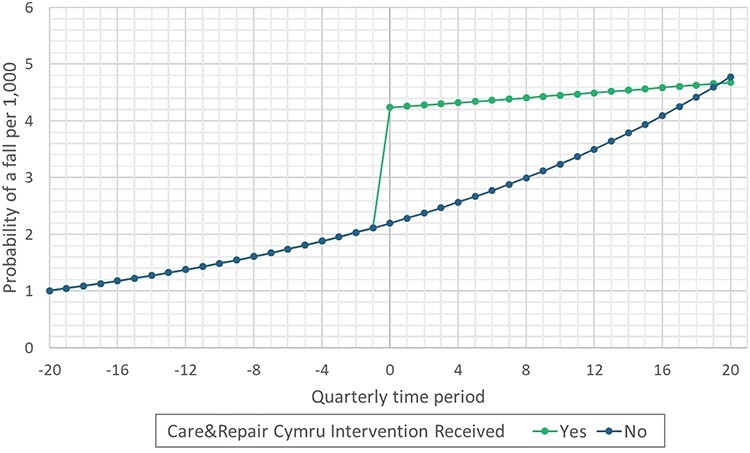
Predicted probability per 1,000 people for a fall at home resulting in an emergency department or hospital admission for Care&Repair clients (green) versus not (blue). The model intercept, time, Care&Repair status, Care&Repair status interaction with time, gender (female), eFI (Mild), WIMD (3) and age (fixed at 65) were included in the probability calculation.

The multilevel logistic regression results for the multivariate model are presented in Table 2 and graphically in Figure 2. The predicted probability of a fall at home resulting in an ED or hospital admission is included in Figure 3, results are displayed as falls per 1,000 people. We have included the stepwise models in the Supplementary material, Supplementary Tables S6 and S7. The model coefficients, odds ratios (ORs) and 95% confidence intervals are recorded for each variable. A caterpillar plot for the regional residuals from the null model is included in the Supplementary material, Supplementary Figure S1.

### Falls odds

Results showed the odds of a fall increased over time, with an OR of 1.04 (1.039, 1.040) per quarter (approximate OR of 1.17 per year). The results also indicated the odds of a fall increased for more deprived areas, increased age, sex (female) and increased frailty (eFI). Compared to those identified as fit, there was increased odds of a fall for people with mild, moderate and severe frailty; ORs 1.57 (1.55, 1.60), 2.31 (2.26, 2.35) and 3.04 (2.96, 3.13), respectively.

### C&RC intervention

Results indicate C&R clients had an overall higher odds of falling, with an OR of 1.93 (1.87, 2.00). However, the interaction term for C&RC-clients and the quarterly time periods indicated a reduced odds of falling in the post-intervention period, with an OR of 0.97 (0.96, 0.97) per quarter (approximate OR of 0.87 per year). This reduction in the odds of falling is depicted in Figure 3.

### Intervention sub-types

The ORs for the intervention sub-types indicate a reduced odds of falling for individuals with interventions to prevent falls between levels; OR 0.91 (0.86, 0.96), and interventions to prevent cold homes; OR 0.72 (0.59, 0.89). There was an increased odds for individuals with interventions to prevent falls on a level OR 1.25 (1.20, 1.31). Interventions to prevent falls in bathrooms & bedrooms and on the stairs indicated an increased odds, ORs 1.04 (0.95, 1.14) and 1.03 (0.97, 1.09) respectively, but were statistically insignificant. The interaction terms generally indicated a small increased odds of falls over time for each intervention type.

### Variation at the individual and regional level

The random effects terms indicate the majority of residual variance for falls is at the individual level. However, the amount of variation per region is still statistically significant, this can be seen further in the regional level residuals in Supplementary Figure S1. The estimated variance partition coefficients (see VPC supplementary for the calculation) indicated ~31.3% and 5.8% of the variation was at the individual and C&RC regional level, respectively.

## Discussion

The descriptive data highlight differences between the C&RC clients and non-clients. The C&RC client group had a higher proportion of people admitted to ED or hospital with a fall, more females, increased severity of frailty, older age and more people living in deprived areas. This indicates that C&RC are targeting a more vulnerable sub-population of older adults, likely consistent with the reactive method of referral to the service, for example, after an older person has experienced a fall. This service profile relating to ‘secondary falls prevention’ might well relate to service capacity limitations and the emphasis of statutory services (as referrer/commissioner) on reactive intervention. This also raises the question of the need for greater capacity to address primary prevention in those who are likely to be at risk of a fall in the future.

The peak in the rate of falls at the index date could indicate that C&RC are reacting to provide services to those that are admitted to ED or hospital. Following the index date, the rate of fallers for non-C&RC clients continues to increase. The rate of fallers for C&RC, however, decreased for 5 quarters (15 months), and then began to increase. This indicates the benefits of C&RC services are most prevalent in the immediate five quarters following a fall related A&E or emergency hospital admission. This reduction in odds following a fall is important in terms of post-hospital discharge recuperation and the stability required to introduce support for rehabilitation and rehabilitation services. In particular, the first year of recovery after a fall is where independence is threatened the most.

The plots stratified by region indicate differences in the rate of falls per region. This highlights the variation between regions and consequently the need for regional differences in service provision. Understanding regional variation is important as there is a ‘post-code lottery’ of local funding to support adaptations and other relevant falls prevention solutions. Also, the regional service’s relationship with NHS and Social Care has implications for the ability to target resources for those most in need.

Consistent with an increasing risk of falls with increasing age the regression model identified an increased odds of falling with time (1.04 [1.04, 1.04], ~1.17 per year). The model also indicated a higher odds of falls for C&RC clients (C&R-client; OR 1.93 [1.87, 2.00]). However, the interaction term for time with C&RC intervention status indicated a reduction in the trajectory for the odds of falls over time (C&R-client time interaction; OR 0.97 [0.96, 0.97] per quarter, ~0.87 per year). This indicates that although C&RC cannot prevent falls, the probability for subsequent falls has a reduced rate. Figure 3 highlights this with a reduction in the gradient of the slope after the Care&Repair service has been received. This is an important finding as the risk of falling is greatly increased for individuals with a history of falls [17].

There was a reduced odds of falling for those receiving interventions for cold prevention and to prevent falls between levels, but an increase in odds for interventions for falls on a level. This could represent the proactive (interventions with reduced odds) versus reactive (increased odds) nature of how and when these interventions are supplied. The interaction terms for specific interventions with time did not show a significant reduction in the odds of falls over time, this finding together with the decreased OR for the C&RC interaction term suggests that the C&RC service is likely to be most effective when combining interventions. This coincides with C&RC’s mission statement to actively work to ensure that all older people have homes that are safe, secure and appropriate to their needs. Similarly, the model shows the majority of variability is at the individual level rather than per region; indicating that the probability of a fall is influenced more by the individual than the area.

### Strengths

The ability to link together administrative, health, and geographic information was a strength of the study. This enabled us to include demographic details as well as information on interventions, frailty and social economic status. We were able to create a large longitudinal cohort of older adults and include national level interventions. Using the linked longitudinal data, we were able to mitigate recall bias and create extended follow-up periods of up to 5-years. This enabled us to quantitatively evaluate how the C&RC service, and specific intervention groups, impacted the odds of falls for older adults in Wales.

### Limitations

We acknowledge that the large power afforded by the sample size may have led to trivial differences being identified as statistically significant. We created a longitudinal dataset, which included the region as a time-varying covariate; however, we did not censor individuals for address changes. This may influence the results where individuals have been required to move home due to increased dependence which may include falls risks. As with all retrospective data linkage studies, there may be errors due to poor coding and recording of outcomes; we anticipate this is a small risk in our study due to the large number of individuals included. Unfortunately, we were unable to include the quarterly time periods as fixed effects in our analysis due to the large number of additional variables and complexity this would create. The eFI contains a deficit for falls, we acknowledge this may interact with our main outcome, but note that the falls recorded in primary care data would not be of the same severity as those recorded as our primary outcome; fall-related emergency attendance at an ED or hospital.

### Further work

For future studies, we would advise matching the C&RC clients to non-clients based on the history of prior falls rather than including a random intercept for each individual. This would simplify the analysis and interpretation significantly and would attempt to further address the potential health needs bias and to better control for unexplained differences between the two groups. The inclusion of paramedic and ambulance data would also be beneficial for emergency falls where an admission to ED or hospital was not required. We would also aim to include an economic analysis for the cost–benefit of the C&RC service.

## Conclusion

Care & Repair services are currently highly mobilised around critical and reactive service delivery, supporting a statutory, and health sector that is struggling to keep up with age-related demand trends. Genuine prevention is not yet fully realisable as a commissioned service model, and yet it is here that the unlocking of long-term transformative savings could lie. There are already positive trends that women are more likely to take up a Care & Repair service, and also that demand pressures are focussed from a severely frail client group. From a long-term policy perspective, it would be worth considering building additional capacity within the model to address early evidence of need, amongst the social priority groupings, that face a risk of falling. Thus, provide preventative interventions earlier in the life course, to those that pose greatest circumstantial risk as life transitions occur.

## Supplementary Material

aa-21-0936-File002_afab201
