# Supplementary material for: Do home adaptation interventions help to reduce emergency fall admissions? A national longitudinal data-linkage study of 657,536 older adults living in Wales (UK) between 2010 and 2017
Source: Age Ageing. 2021 Oct 18;51(1):afab201. doi: 10.1093/ageing/afab201 (PMC8753038; doi:10.1093/ageing/afab201)
Supplement: aa-21-0936-File002_afab201 [file aa-21-0936-file002_afab201.docx]

**Do home adaptation interventions help to reduce emergency fall admissions? A national longitudinal data-linkage study of 657,536 older adults living in Wales (UK) between 2010-2017.**

**Contents**

Care&Repair Cymru Interventions*, page 2*

Falls coding, *page 3*

Care&Repair Cymru Regions in relation to Welsh Local Authorities, *page4*

Extended descriptive data for the sub-populations*, page 5*

Stepwise multilevel logistic regression models*, page 8*

Variance Partition Coefficient for the individual and regional level, *page 10*

Care&Repair Cymru Regional Residuals*, page 11*

**Care&Repair Cymru Interventions**

Table S1. Care&Repair Cymru Specified Interventions

| Category | Interventions | Count (% of total clients) |
| --- | --- | --- |
| **Care&Repair Clients** |  | 123,729 |
| **Falls on a level** | 12" Grab Rail, 18" Grab Rail, 24" Grab Rail, Grab Rails Carpet tacking/Re-gripping, Cranked rail, Exterior Lighting, Floor Coverings, Flooring, Lightbulbs, Lighting – additional, Bed/Chair Raisers, Fit Only Grab Rails, Hand Rails, Drop Down Rails, Level Threshold, Moving Furniture, Non-slip surface, Outside Lighting, Telecare, Curtain Rails, Curtains, Doors – widen, Driveway, Gutters- clearing, Paths, Shelves, Floor Coverings, Floor Levelling, Floor markings, Floor to ceiling pole, Leak repair, Guttering, Gutters – repairing, Gutters – replacing, Leaks, Drop curb | 72,151 (58.3%) |
| **Falls on stairs** | Bannister, Stair Rail, Stairlift, Through Floor Lift, Newel Rails, Redesign Staircase, Stairlift repair, Newel Posts, Safety Gates | 27,595 (22.3%) |
| **Falls between levels** | Step Lift, External Rails, Temporary Ramp, Ramps, Half Step, Full Step, Steps, Medway Rails | 27,349 (22.1%) |
| **Falls in bathroom/bedroom** | Level Access Shower, Shower alterations, Shower Seats, Shower Tray, Toilet Frames, Swedish bath rail, Bathroom Redesign, W.C. Redesign, W.C. – additional facility, Bath/Shower Steps, Hoist, Raised toilet seat/bidet, Ceiling track hoist, Bathroom repairs, Shower - over bath, Shower screens, Closomat | 11,716 (9.5%) |
| **Cold Homes (*indirect)** | Boiler repairs, Central Heating, Heating Repairs, Replace Boiler, Cavity Wall Insulation, Draught Proofing, Electric Heating | 3,201 (2.6%) |

**Falls coding**

| Outcome , falls at home defined as: |
| --- |
| Patient Episode Database for Wales |
| (EPISODE.DIAG_CD_123 BETWEEN 'S00' AND 'T14') --restrict to traumatic injuries |
| AND DIAG.DIAG_CD_123 IN ('W01','W03','W04','W05','W06','W07','W08','W10','W11','W12','W13','W17','W18','W19') --falls codes |
| AND DIAG.DIAG_CD_4 = '0' --falls at home |
| AND diag.epi_num = 1 --episode number = 1 |
| AND spell.ADMIS_MTHD_CD LIKE '2%'-- emergency admissions only |
|  |
| Emergency Department DataSet |
| mech_of_inj = '01' --specifies a fall/trip/slip |
| and attend_category = '01' --specifies a new visit |
| and location_type = '01' --Specify home as location type |
|  |

**Care&Repair Cymru Regions in relation to Welsh Local Authorities**

Table S2. Care & Repair Cymru region composition in relation to Welsh Local Authorities

| C&RC Region | Local Authorities | LSOA 2011 count (total = 1909) |
| --- | --- | --- |
| Blaneau Gwent and Caerphilly | Blaneau Gwent | 47 |
|  | Caerphilly | 110 |
| Bridgend County | Bridgend | 88 |
| Cardiff and the Vale | Cardiff | 214 |
|  | The Vale of Glamorgan | 79 |
| Carmarthenshire | Carmarthenshire | 112 |
| North East Wales | Flintshire | 92 |
|  | Wrexham | 85 |
| Western Bay | Neath Port Talbot | 91 |
|  | Swansea | 148 |
| Conwy and Denbighshire | Conwy | 71 |
|  | Denbighshire | 58 |
| Cwm Taf | Merthyr Tydfil | 36 |
|  | Rhondda Cynon Taf | 154 |
| Gofal a Thrwsio Gwynedd a Môn | Gwynedd | 73 |
|  | Isle of Anglesey | 44 |
| Monmouthshire and Torfaen | Monmouthshire | 56 |
|  | Torfaen | 60 |
| Newport | Newport | 95 |
| Powys | Powys | 79 |
| West Wales | Ceredigion | 46 |
|  | Pembrokeshire | 71 |

**Extended descriptive data for the sub-populations**

Table S3. Extended descriptive data for the combined cohort

| Combined |  |  |  |  |  |  |  |  |  |  |  |  |  |  |  |  |  |
| --- | --- | --- | --- | --- | --- | --- | --- | --- | --- | --- | --- | --- | --- | --- | --- | --- | --- |
| Quarter | -20 | -15 | -10 | -5 | -4 | -3 | -2 | -1 | 0 | 1 | 2 | 3 | 4 | 5 | 10 | 15 | 20 |
| Individuals (N) | 633686 | 639122 | 644505 | 650204 | 651441 | 652706 | 654171 | 655671 | 657536 | 628487 | 602970 | 578641 | 553891 | 531039 | 427353 | 331532 | 243294 |
| Falls (N) | 897 | 1318 | 1735 | 2324 | 2556 | 2951 | 3928 | 9858 | 4758 | 4368 | 4191 | 3971 | 3841 | 3650 | 2927 | 2351 | 1822 |
| Falls rate | 0.14% | 0.21% | 0.27% | 0.36% | 0.39% | 0.45% | 0.60% | 1.50% | 0.72% | 0.70% | 0.70% | 0.69% | 0.69% | 0.69% | 0.68% | 0.71% | 0.75% |
| Mean Age | 72.05 | 72.03 | 72.02 | 72 | 72 | 72 | 72 | 72 | 72 | 71.87 | 71.75 | 71.64 | 71.53 | 71.42 | 70.87 | 70.33 | 69.81 |
| s.d. Age | 8.79 | 8.78 | 8.79 | 8.79 | 8.79 | 8.79 | 8.79 | 8.79 | 8.8 | 8.74 | 8.68 | 8.62 | 8.56 | 8.51 | 8.21 | 7.9 | 7.6 |
| Gender - Male | 46.18% | 46.21% | 46.24% | 46.25% | 46.26% | 46.26% | 46.25% | 46.25% | 46.25% | 46.14% | 46.06% | 45.99% | 45.92% | 45.87% | 45.62% | 45.47% | 45.39% |
| Gender - Female | 53.82% | 53.79% | 53.76% | 53.75% | 53.74% | 53.74% | 53.75% | 53.75% | 53.75% | 53.86% | 53.94% | 54.01% | 54.08% | 54.13% | 54.38% | 54.53% | 54.61% |
| eFI - Fit | 68.95% | 64.38% | 60.25% | 56.56% | 55.87% | 55.16% | 54.43% | 53.68% | 52.70% | 52.33% | 52.02% | 51.70% | 51.38% | 51.07% | 49.85% | 48.88% | 48.07% |
| eFI - Mild | 25.32% | 27.97% | 30.03% | 31.55% | 31.80% | 32.06% | 32.30% | 32.48% | 32.66% | 32.75% | 32.83% | 32.94% | 33.10% | 33.23% | 33.79% | 34.42% | 35.04% |
| eFI - Moderate | 5.08% | 6.65% | 8.25% | 9.84% | 10.15% | 10.45% | 10.79% | 11.18% | 11.73% | 11.89% | 12.04% | 12.16% | 12.24% | 12.37% | 12.75% | 13.07% | 13.24% |
| eFI - Severe | 0.66% | 1.00% | 1.47% | 2.05% | 2.18% | 2.33% | 2.48% | 2.65% | 2.91% | 3.03% | 3.11% | 3.20% | 3.27% | 3.34% | 3.60% | 3.63% | 3.65% |
| WIMD - 1.Least Deprived | 22.49% | 22.49% | 22.49% | 22.50% | 22.49% | 22.49% | 22.49% | 22.49% | 22.49% | 22.51% | 22.53% | 22.57% | 22.60% | 22.60% | 22.78% | 22.94% | 23.11% |
| 2 | 19.55% | 19.60% | 19.67% | 19.73% | 19.74% | 19.74% | 19.76% | 19.78% | 19.80% | 19.83% | 19.82% | 19.79% | 19.79% | 19.79% | 19.79% | 19.81% | 19.87% |
| 3 | 21.01% | 21.08% | 21.09% | 21.11% | 21.12% | 21.13% | 21.13% | 21.13% | 21.14% | 21.14% | 21.16% | 21.16% | 21.13% | 21.15% | 21.10% | 21.09% | 21.05% |
| 4 | 19.78% | 19.74% | 19.73% | 19.72% | 19.72% | 19.72% | 19.72% | 19.72% | 19.71% | 19.69% | 19.68% | 19.66% | 19.67% | 19.66% | 19.64% | 19.54% | 19.52% |
| 5.Most Deprived | 17.17% | 17.10% | 17.01% | 16.94% | 16.94% | 16.92% | 16.90% | 16.88% | 16.86% | 16.83% | 16.82% | 16.82% | 16.81% | 16.79% | 16.70% | 16.61% | 16.46% |

Table S4. Extended descriptive data for individuals not receiving an intervention from Care&Repair.

| Non-clients |  |  |  |  |  |  |  |  |  |  |  |  |  |  |  |  |  |
| --- | --- | --- | --- | --- | --- | --- | --- | --- | --- | --- | --- | --- | --- | --- | --- | --- | --- |
| Quarter | -20 | -15 | -10 | -5 | -4 | -3 | -2 | -1 | 0 | 1 | 2 | 3 | 4 | 5 | 10 | 15 | 20 |
| Individuals (N) | 513427 | 518169 | 522911 | 527906 | 528988 | 530065 | 531317 | 532492 | 533807 | 514012 | 495180 | 476621 | 457443 | 439488 | 357527 | 280313 | 207647 |
| Falls (N) | 619 | 942 | 1213 | 1498 | 1669 | 1826 | 1938 | 2025 | 2202 | 2189 | 2212 | 2097 | 2043 | 1975 | 1643 | 1344 | 1119 |
| Falls rate | 0.12% | 0.18% | 0.23% | 0.28% | 0.32% | 0.34% | 0.36% | 0.38% | 0.41% | 0.43% | 0.45% | 0.44% | 0.45% | 0.45% | 0.46% | 0.48% | 0.54% |
| Mean Age | 70.64 | 70.63 | 70.62 | 70.6 | 70.6 | 70.6 | 70.6 | 70.6 | 70.6 | 70.5 | 70.41 | 70.32 | 70.23 | 70.14 | 69.68 | 69.25 | 68.82 |
| s.d. Age | 8.24 | 8.24 | 8.24 | 8.24 | 8.24 | 8.24 | 8.25 | 8.25 | 8.25 | 8.18 | 8.12 | 8.06 | 8 | 7.94 | 7.64 | 7.35 | 7.07 |
| Gender - Male | 48.16% | 48.18% | 48.20% | 48.22% | 48.22% | 48.22% | 48.22% | 48.21% | 48.22% | 48.21% | 48.20% | 48.17% | 48.15% | 48.13% | 48.04% | 47.97% | 47.93% |
| Gender - Female | 51.84% | 51.82% | 51.80% | 51.78% | 51.78% | 51.78% | 51.78% | 51.79% | 51.78% | 51.79% | 51.80% | 51.83% | 51.85% | 51.87% | 51.96% | 52.03% | 52.07% |
| eFI - Fit | 73.34% | 69.17% | 65.31% | 61.87% | 61.22% | 60.59% | 59.94% | 59.34% | 58.71% | 58.30% | 57.93% | 57.55% | 57.16% | 56.81% | 55.24% | 53.86% | 52.69% |
| eFI - Mild | 22.42% | 25.15% | 27.44% | 29.24% | 29.55% | 29.84% | 30.14% | 30.38% | 30.65% | 30.91% | 31.11% | 31.35% | 31.62% | 31.83% | 32.85% | 33.77% | 34.58% |
| eFI - Moderate | 3.79% | 4.99% | 6.23% | 7.48% | 7.72% | 7.97% | 8.22% | 8.49% | 8.74% | 8.85% | 8.99% | 9.11% | 9.20% | 9.31% | 9.76% | 10.18% | 10.51% |
| eFI - Severe | 0.45% | 0.68% | 1.02% | 1.41% | 1.50% | 1.60% | 1.69% | 1.80% | 1.91% | 1.94% | 1.96% | 1.99% | 2.02% | 2.05% | 2.15% | 2.19% | 2.22% |
| WIMD - 1.Least Deprived | 23.20% | 23.20% | 23.19% | 23.19% | 23.18% | 23.18% | 23.18% | 23.18% | 23.17% | 23.19% | 23.21% | 23.28% | 23.32% | 23.34% | 23.58% | 23.77% | 23.96% |
| 2 | 19.62% | 19.67% | 19.74% | 19.80% | 19.81% | 19.82% | 19.83% | 19.86% | 19.87% | 19.89% | 19.89% | 19.87% | 19.86% | 19.87% | 19.87% | 19.89% | 19.94% |
| 3 | 20.81% | 20.88% | 20.90% | 20.92% | 20.94% | 20.95% | 20.95% | 20.95% | 20.96% | 20.95% | 20.97% | 20.97% | 20.95% | 20.95% | 20.88% | 20.85% | 20.81% |
| 4 | 19.30% | 19.26% | 19.25% | 19.25% | 19.24% | 19.24% | 19.25% | 19.24% | 19.23% | 19.22% | 19.21% | 19.19% | 19.19% | 19.17% | 19.13% | 19.04% | 19.01% |
| 5.Most Deprived | 17.08% | 17.00% | 16.91% | 16.83% | 16.82% | 16.81% | 16.78% | 16.77% | 16.77% | 16.74% | 16.71% | 16.69% | 16.68% | 16.67% | 16.53% | 16.44% | 16.27% |

Table S5. Extended descriptive data for the Care&Repair cohort .

| Care&Repair clients |  |  |  |  |  |  |  |  |  |  |  |  |  |  |  |  |  |
| --- | --- | --- | --- | --- | --- | --- | --- | --- | --- | --- | --- | --- | --- | --- | --- | --- | --- |
|  | -20 | -15 | -10 | -5 | -4 | -3 | -2 | -1 | 0 | 1 | 2 | 3 | 4 | 5 | 10 | 15 | 20 |
| Individuals (N) | 120259 | 120953 | 121594 | 122298 | 122453 | 122641 | 122854 | 123179 | 123729 | 114475 | 107790 | 102020 | 96448 | 91551 | 69826 | 51219 | 35647 |
| Falls (N) | 278 | 376 | 522 | 826 | 887 | 1125 | 1990 | 7833 | 2556 | 2179 | 1979 | 1874 | 1798 | 1675 | 1284 | 1007 | 703 |
| Falls rate | 0.23% | 0.31% | 0.43% | 0.68% | 0.72% | 0.92% | 1.62% | 6.36% | 2.07% | 1.90% | 1.84% | 1.84% | 1.86% | 1.83% | 1.84% | 1.97% | 1.97% |
| Mean Age | 78.07 | 78.06 | 78.05 | 78.04 | 78.04 | 78.04 | 78.03 | 78.03 | 78.04 | 77.99 | 77.9 | 77.78 | 77.67 | 77.58 | 76.97 | 76.28 | 75.61 |
| s.d. Age | 8.5 | 8.5 | 8.51 | 8.51 | 8.51 | 8.51 | 8.52 | 8.52 | 8.52 | 8.5 | 8.48 | 8.47 | 8.45 | 8.43 | 8.31 | 8.17 | 8.01 |
| Gender - Male | 37.75% | 37.74% | 37.77% | 37.76% | 37.77% | 37.77% | 37.76% | 37.76% | 37.77% | 36.85% | 36.24% | 35.77% | 35.30% | 35.00% | 33.27% | 31.82% | 30.60% |
| Gender - Female | 62.25% | 62.26% | 62.23% | 62.24% | 62.23% | 62.23% | 62.24% | 62.24% | 62.23% | 63.15% | 63.76% | 64.23% | 64.70% | 65.00% | 66.73% | 68.18% | 69.40% |
| eFI - Fit | 50.20% | 43.88% | 38.47% | 33.64% | 32.74% | 31.72% | 30.61% | 29.25% | 26.77% | 25.53% | 24.86% | 24.39% | 23.95% | 23.51% | 22.29% | 21.63% | 21.19% |
| eFI - Mild | 37.68% | 40.04% | 41.16% | 41.54% | 41.54% | 41.62% | 41.61% | 41.57% | 41.32% | 41.01% | 40.73% | 40.37% | 40.12% | 39.92% | 38.61% | 37.99% | 37.67% |
| eFI - Moderate | 10.59% | 13.74% | 16.92% | 20.04% | 20.62% | 21.19% | 21.91% | 22.84% | 24.65% | 25.51% | 26.03% | 26.42% | 26.69% | 27.03% | 28.06% | 28.90% | 29.13% |
| eFI - Severe | 1.53% | 2.35% | 3.44% | 4.78% | 5.11% | 5.47% | 5.86% | 6.35% | 7.26% | 7.94% | 8.39% | 8.83% | 9.23% | 9.54% | 11.04% | 11.48% | 12.00% |
| WIMD - 1.Least Deprived | 19.47% | 19.47% | 19.47% | 19.49% | 19.50% | 19.50% | 19.51% | 19.51% | 19.53% | 19.44% | 19.36% | 19.26% | 19.16% | 19.08% | 18.66% | 18.39% | 18.13% |
| 2 | 19.29% | 19.31% | 19.36% | 19.39% | 19.40% | 19.42% | 19.44% | 19.45% | 19.51% | 19.56% | 19.49% | 19.44% | 19.49% | 19.42% | 19.37% | 19.38% | 19.41% |
| 3 | 21.87% | 21.90% | 21.93% | 21.92% | 21.91% | 21.90% | 21.92% | 21.92% | 21.94% | 21.98% | 22.02% | 22.03% | 22.00% | 22.13% | 22.20% | 22.40% | 22.41% |
| 4 | 21.82% | 21.81% | 21.76% | 21.77% | 21.77% | 21.77% | 21.76% | 21.78% | 21.75% | 21.77% | 21.80% | 21.86% | 21.95% | 22.02% | 22.25% | 22.28% | 22.47% |
| 5.Most Deprived | 17.56% | 17.51% | 17.47% | 17.42% | 17.42% | 17.41% | 17.38% | 17.34% | 17.27% | 17.25% | 17.33% | 17.41% | 17.40% | 17.36% | 17.52% | 17.56% | 17.58% |

**Stepwise multilevel logistic regression models**

Table S6. Model coefficients for the stepwise multilevel logistic regression models.

| *Coefficients* |  |  |  |  |  |  |
| --- | --- | --- | --- | --- | --- | --- |
| Intercept | -5.274 (-5.562,-4.986) | -5.431 (-5.716,-5.146) | -11.458 (-11.768,-11.149) | -11.94 (-12.25,-11.629) | -11.206 (-11.516,-10.895) | -11.34 (-11.646,-11.034) |
| Care&Repair Client |  | 1.215 (1.19,1.239) | 0.847 (0.824,0.871) | 0.838 (0.814,0.861) | 0.742 (0.719,0.765) | 0.742 (0.719,0.765) |
| Time Period (Quarter) |  | 0.035 (0.035,0.036) | 0.044 (0.043,0.045) | 0.044 (0.043,0.045) | 0.039 (0.038,0.039) | 0.039 (0.038,0.04) |
| Time Period (Quarter):Care&Repair Client |  | -0.02 (-0.022,-0.017) | -0.023 (-0.025,-0.021) | -0.025 (-0.027,-0.022) | -0.027 (-0.03,-0.025) | -0.027 (-0.03,-0.025) |
| Age |  |  | 0.081 (0.08,0.082) | 0.079 (0.078,0.08) | 0.065 (0.064,0.066) | 0.066 (0.065,0.066) |
| Gender (Female) |  |  |  | 0.405 (0.39,0.42) | 0.364 (0.349,0.379) | 0.364 (0.349,0.379) |
| eFI-Mild |  |  |  |  | 0.458 (0.442,0.475) | 0.453 (0.436,0.47) |
| eFI-Moderate |  |  |  |  | 0.849 (0.83,0.869) | 0.84 (0.82,0.86) |
| eFI-Severe |  |  |  |  | 1.133 (1.105,1.161) | 1.121 (1.093,1.149) |
| WIMD (2014) |  |  |  |  |  | 0.039 (0.034,0.044) |
| *Random effects* | - | - | - | - | - | - |
| Individual level variance | 3.356 (3.324,3.387) | 3.133 (3.101,3.164) | 2.094 (2.067,2.121) | 2.046 (2.019,2.073) | 1.638 (1.614,1.663) | 1.653 (1.629,1.678) |
| Care&Repair level variance | 0.281 (0.065,0.497) | 0.275 (0.063,0.486) | 0.311 (0.072,0.55) | 0.312 (0.072,0.552) | 0.311 (0.072,0.55) | 0.301 (0.069,0.532) |
| - | - | - | - | - | - | - |
| Observations | 22016986 | 22016986 | 22016986 | 22016986 | 22016986 | 22016986 |
| Individuals | 657536 | 657536 | 657536 | 657536 | 657536 | 657536 |
| Care&Repair Regions | 13 | 13 | 13 | 13 | 13 | 13 |
| AUC | 0.5 | 0.669 | 0.749 | 0.754 | 0.771 | 0.772 |
| AUC 95% CI |  | 0.6673-0.6706 | 0.7472-0.7503 | 0.7527-0.7557 | 0.7691-0.7719 | 0.7705-0.7734 |

Table S7. Odds Ratios for the stepwise multilevel logistic regression models.

| *Odds Ratios* |  |  |  |  |  |  |
| --- | --- | --- | --- | --- | --- | --- |
| Intercept | 0.005 (0.004,0.007) | 0.004 (0.003,0.006) | 0 (0,0) | 0 (0,0) | 0 (0,0) | 0 (0,0) |
| Care&Repair Client |  | 3.369 (3.287,3.454) | 2.334 (2.28,2.389) | 2.311 (2.258,2.365) | 2.1 (2.052,2.149) | 2.101 (2.053,2.15) |
| Time Period (Quarter) |  | 1.036 (1.035,1.037) | 1.045 (1.044,1.046) | 1.045 (1.044,1.046) | 1.04 (1.039,1.04) | 1.04 (1.039,1.04) |
| Time Period (Quarter):Care&Repair Client |  | 0.981 (0.978,0.983) | 0.977 (0.975,0.979) | 0.976 (0.974,0.978) | 0.973 (0.971,0.975) | 0.973 (0.971,0.975) |
| Age |  |  | 1.085 (1.084,1.086) | 1.082 (1.081,1.083) | 1.068 (1.067,1.068) | 1.068 (1.067,1.069) |
| Gender (Female) |  |  |  | 1.5 (1.477,1.523) | 1.439 (1.418,1.461) | 1.439 (1.417,1.46) |
| eFI-Mild |  |  |  |  | 1.582 (1.556,1.608) | 1.573 (1.547,1.6) |
| eFI-Moderate |  |  |  |  | 2.338 (2.292,2.385) | 2.316 (2.27,2.363) |
| eFI-Severe |  |  |  |  | 3.105 (3.019,3.193) | 3.068 (2.983,3.156) |
| WIMD (2014) |  |  |  |  |  | 1.04 (1.034,1.045) |
| *Random effects* | - | - | - | - | - | - |
| Individual level variance | 3.356 (3.324,3.387) | 3.133 (3.101,3.164) | 2.094 (2.067,2.121) | 2.046 (2.019,2.073) | 1.638 (1.614,1.663) | 1.653 (1.629,1.678) |
| Care&Repair level variance | 0.281 (0.065,0.497) | 0.275 (0.063,0.486) | 0.311 (0.072,0.55) | 0.312 (0.072,0.552) | 0.311 (0.072,0.55) | 0.301 (0.069,0.532) |
| - | - | - | - | - | - | - |
| Observations | 22016986 | 22016986 | 22016986 | 22016986 | 22016986 | 22016986 |
| Individuals | 657536 | 657536 | 657536 | 657536 | 657536 | 657536 |
| Care&Repair Regions | 13 | 13 | 13 | 13 | 13 | 13 |

**VPC Supplementary: Variance Partition Coefficient for the individual and regional level**

We used the latent variable method to estimate the variance partition coefficient at the individual (VPC_Ind_) and regional (VPC_C&RC_) levels. The estimations were calculated as follows:

$VPC_{C\&RC}={\sigma_{C\&RC}}^{2}/{{(\sigma}_{C\&RC}}^{2}+{\sigma_{Ind}}^{2}+\pi^{2}/3)= 0.302/(0.302+1.635+ \pi^{2}/3)= 0.058$

$VPC_{Ind} ={\sigma_{Ind}}^{2}/{{(\sigma}_{C\&RC}}^{2}+{\sigma_{Ind}}^{2}+\pi^{2}/3)= 1.635/(0.302+1.635+ \pi^{2}/3)= 0.313$

**Care&Repair Cymru Regional Residuals**


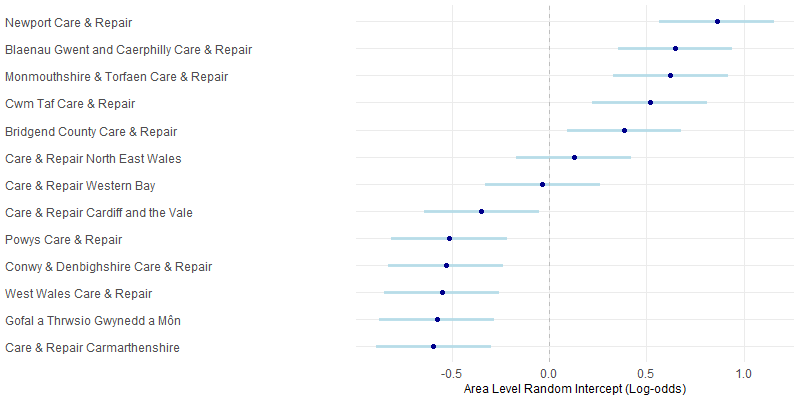
Figure S1. Region level residuals for the null logistic regression model. The null model included an intercept term with random intercepts included at the individual and regional level.
